# RNA substrate length as an indicator of exosome interactions *in vivo*

**DOI:** 10.12688/wellcomeopenres.10724.2

**Published:** 2017-07-03

**Authors:** Clémentine Delan-Forino, Claudia Schneider, David Tollervey

**Affiliations:** 1 Wellcome Trust Centre for Cell Biology, University of Edinburgh, Edinburgh, EH9 3BF, UK; 2 Institute for Cell and Molecular Biosciences, Newcastle University, Newcastle upon Tyne, NE2 4HH, UK

**Keywords:** Exosome, RNA processing, RNA degradation, protein-RNA interaction, RNA-binding sites, UV crosslinking, yeast

## Abstract

*Background: *The exosome complex plays key roles in RNA processing and degradation in Eukaryotes and Archaea. Outstanding structural studies identified multiple pathways for RNA substrates into the exosome *in vitro*, but identifying the pathway followed by individual RNA species *in vivo* remains challenging.

*Methods: *We attempted to address this question using RNase protection. *In vivo* RNA-protein crosslinking (CRAC) was applied to the exosome component Rrp44/Dis3, which has both endonuclease and exonuclease activity. During CRAC, the exosome was purified under native conditions and subjected to RNase digestion, prior to protein denaturation and cDNA cloning. The resulting high-throughput sequence reads were stratified by length of the cDNA sequence. This should reflect RNA fragment lengths, and therefore the RNA region that was protected by exosome binding. We anticipated major read lengths of ~30nt and ~10nt, reflecting the “central channel” and “direct access” routes to the Rrp44 exonuclease active site observed *in vitro*.

*Results:* Unexpectedly, no clear peak was observed at 30nt, whereas a broad peak was seen around 20nt. The expected ~10nt peak was seen, and showed strong elevation in strains lacking exonuclease activity. Unexpectedly, this peak was suppressed by point mutations in the Rrp44 endonuclease active site. This indicates that the short fragments are degraded by the exonuclease activity of Rrp44, but also suggests that at least some may be generated by endonuclease activity.

*Conclusions: *The absence of 30nt protected fragments may reflect obligatory binding of cofactors at the entrance to the exosome central channel *in vivo*. The presence of ~20nt fragments apparently indicates an access route not yet reported from *in vitro* studies. Confident mapping of 10nt reads is challenging, but they are clearly derived from a subset of exosome targets. In particular, pre-rRNA species, which are major exosome targets, are strongly disfavored for the generation of short reads.

## Introduction

**Figure 1.**
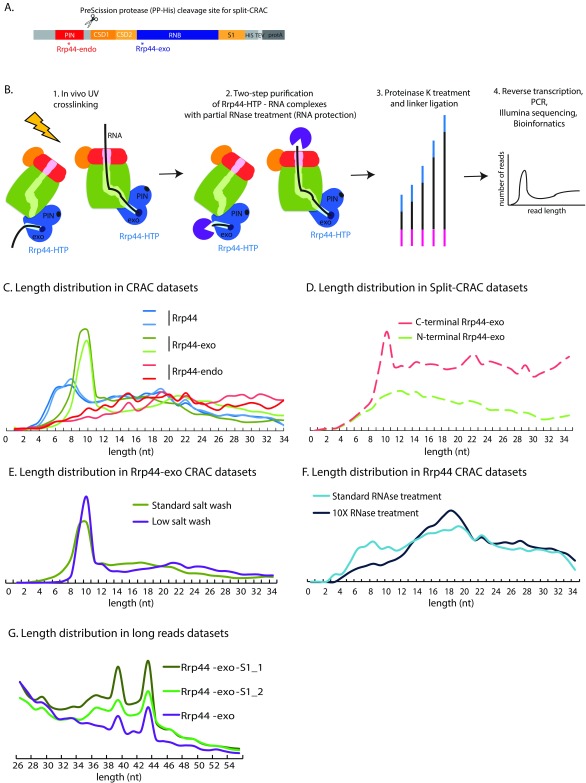
Exosome structure and interactions. ( **A**) Domain structure of the Rrp44-HTP fusion. From N-terminus to C-terminus, the following domains are indicated: PIN domain harboring endonuclease activity, CSD (Cold-Shock RNA binding domain), RNB (ribonuclease) domain harboring exonuclease activity, S1 RNA binding domain and the HTP-tag (His _6_, TEV protease cleavage site, protein A). Asterisks represent location of point mutations in Rrp44-endo and Rrp44-exo. The PreScission protease cleavage site and associated His6 tag (PP-His) used in split-CRAC is represented as scissors. ( **B**) Overview of the CRAC experiment on Rrp44-HTP. The main components of the exosome are schematically represented: the cap in red, the RNase PH-ring in green. The PIN endonuclease and exonuclease (exo) active sites of Rrp44 are indicated in dark blue. Exponentially growing cells were UV crosslinked (1), RNA associated with Rrp44, either by threading or direct access, was purified via a two-step purification involving partial RNase treatment (2), processed by linker ligation followed by proteinase K digestion (3), reverse-transcribed, PCR amplified and Illumina sequenced (4). ( **C**) Length distribution of reads recovered in CRAC datasets for Rrp44, Rrp44-exo or Rrp44-endo. Two independent experiments for each protein are shown. ( **D**) Length distributions of reads recovered with Rrp44-exo N-terminal and C-terminal regions, obtained by split-CRAC. ( **E**) Length distribution of reads recovered by Rrp44-exo CRAC using either standard salt washes (used for all other CRAC datasets presented in this study, 1M NaCl, green line) or standard salt washes (350nM NaCl, purple line). ( **F**) Length distribution of reads recovered by Rrp44 CRAC using either standard RNase treatment (used for all other CRAC datasets presented in this study, light blue line) or 10X RNase treatment (dark blue line). ( **G**) Length distribution of long reads recovered by Rrp44-exo (purified with 350 nM NaCl, sequenced on 150nt Illumina run) and Rrp44-exo-S1 CRAC (purified with 1M NaCl, sequenced on 100nt Illumina run).

The exosome nuclease complex in Eukaryotes has a barrel-like structure, with a central channel through which substrate RNAs can be threaded to reach the 3’ exonuclease active site of the RNase II related protein Rrp44 (Dis3). Rrp44 is composed of an N-terminal PIN (PilT N terminus) domain with endonuclease activity, two continuous RNA-binding cold-shock domains (CSD domains), an RNB domain carrying the exonuclease active site, and an RNA-binding S1 domain ( Figure 1A). Initial functional analyses of the PIN endonuclease activity of Rrp44 identified only the 7S pre-rRNA and excised 5’ ETS pre-rRNA fragments as targets for cleavage ( Lebreton *et al*., 2008; Schaeffer *et al*., 2009; Schneider *et al*., 2009). This endonuclease activity is well conserved in evolution and it seemed likely that additional targets would emerge. We previously attempted to identify targets for the PIN domain-associated endonuclease activity by *in vivo* RNA-protein crosslinking and sequencing of the resulting cDNA products (CRAC) ( Figure 1B). To allow specific recovery of RNAs associated with the PIN domain, a His _6_ and PreScission protease cleavage site were introduced immediately C-terminal to this region. The intact protein was crosslinked *in vivo* and the PIN domain was then cleaved off and selectively purified *in vitro* during RNA-protein complex purification. Analysis of the associated RNAs revealed that many different RNAs contact the PIN domain ( Schneider *et al*., 2012).

RNAs that are targeted to the exonuclease domain of Rrp44 can follow at least two routes; threading through the central barrel of the exosome complex, or direct access to the active site. However, identifying the substrates that follow each of these pathways *in vivo* is very challenging. These pathways involve distinct conformations of the exosome and would be expected to protect different lengths of the substrate RNA. *In vitro* analyses have confirmed the protection of the 3’ terminal 30-33 nt for RNAs threaded through the channel, whereas only ~9-10 nt might be expected to be protected on the direct access route. The aim of the work reported here was to use this distinction to identify RNA substrates for each pathway.

## Results

### Length distribution of Rrp44-associated RNAs

CRAC was performed on a Rrp44 construct expressed from the endogenous locus and carrying a tripartite C-terminal HTP tag (His _6_ - TEV protease cleavage site – 2 copies of the Z-domain of protein A) ( Figure 1A). Otherwise, plasmid-encoded wildtype Rrp44-HTP expressed from its endogenous promoter was compared to constructs with Rrp44-HTP that lacked exonuclease activity, due to catalytic site point mutation (D _551_N; Rrp44-exo), or lacked endonuclease activity, due to point mutations at each of the four conserved endonuclease active-site amino acids (D _91_N, E _120_Q, D _171_N, D _198_N; Rrp44-endo).

During CRAC analyses ( Figure 1B), bait proteins were UV crosslinked to associated RNAs in actively growing cells and purified under native conditions. This was followed by partial digestion with RNase A + T1, again under native conditions ( Granneman *et al*., 2009). We therefore expect partial protection (“foot-printing”) of the bound RNA by the protein complex. Subsequently, the proteins were denatured by incubation with 6M Guanidinium HCl prior to binding to a nickel affinity column. Following 5’ and 3’ linker ligation and elution with imidazole, proteins were further purified by denaturing SDS polyacrylamide gel electrophoresis (SDS-PAGE), then digested with proteinase K. Associated RNAs were amplified by RT-PCR and identified by Illumina sequencing.

Figure 1C shows a comparison of the length distribution of reads recovered from two independent experiments. Based on *in vitro* analyses, we expected two major length populations; around 30-33 nt from RNAs threaded through the central channel, and around 9-10 nt from RNAs that directly access the Rrp44 exonuclease site ( Bonneau *et al*., 2009). Surprisingly, the expected ~30 nt fragment peak was not clearly seen for HTP-tagged, catalytically active Rrp44 (Rrp44; blue lines in Figure 1C). Instead, read lengths for wildtype were broadly distributed, but with a clear increase at very short lengths (6-9 nt). In addition, a broader region around 20 nt was elevated.

It seemed possible that the lack of clear 30 nt and 10 nt peaks reflected partial digestion of substrate RNAs by Rrp44 exonuclease activity during the extended incubations needed for RNA purification prior to cDNA generation. We therefore repeated the analysis using Rrp44-exo (green lines in Figure 1C). This also failed to generate a clear 30 nt peak, but did show a broad maximum around 20 nt, together with a dramatically increased peak of reads at 10 nt.

The peak seen in the Rrp44-exo dataset would be consistent with direct access, however, it also seemed possible that the endonuclease activity might generate these fragments by cleavage of substrates, either in the central channel or otherwise docked onto the exosome. We therefore also analyzed an Rrp44-endo mutant strain (red lines in Figure 1C). Strikingly, this mutation almost completely abolished recovery of the short reads seen with wildtype Rrp44 and Rrp44-exo.

In principle, the short, endonuclease-generated RNA fragments could be associated with either the N-terminal PIN domain or C-terminal exonuclease domain of Rrp44. To assess this, we made use of a construct in which a PreScission protease cleavage site, in combination with a His _6_ affinity tag, was introduced into Rrp44-exo at a site C-terminal to the PIN domain ( Figures 1A and D) ( Schneider *et al*., 2012). This allows *in vivo* crosslinking with intact Rrp44-exo, followed by separation of the N-terminal and C-terminal fragments by *in vitro* cleavage during purification. Two constructs were compared in which the His _6_ tag is associated with either the Rrp44 NTD (N-terminal Rrp44-exo; red line in Figure 1D) or CTD (C-terminal Rrp44-exo; green line in Figure 1D) allowing their selective recovery. Comparison of the datasets clearly showed the peak of 10 nt fragments to be associated with the C-terminal domain, which includes the 2 CSD and 1 S1 RNA binding domains, as well as the exonuclease domain.

Together, these data indicate that the C-terminal domain of Rrp44 binds short, ~10 nt RNA fragments that are generated by the endonuclease activity. This suggests the possibility that the endonuclease activity acts to release substrates that are blocked in the exosome channel extending to the Rrp44 exonuclease RNA-binding cleft. These might arise quite frequently because the Rrp44 exonuclease active site is predicted to be highly processive ( Frazão *et al*., 2006; Lorentzen *et al*., 2008), implying the ability to retain and “pull” on substrate RNAs. However, double-stranded regions are unable to enter the central channel of the exosome, potentially blocking further substrate movement.

We considered the possibility that the standard 1M NaCl buffer used for IgG binding and wash might adversely affect the core exosome structure, although previous analyses have indicated substantial salt resistance ( Allmang *et al*., 1999). To assess, we compared the exosome purified using 1M Na Cl (standard salt in Figure 1E) or 350mM NaCl buffer (low salt in Figure 1E), which was generally used in previous purifications of the exosome for structural analyses ( Kowalinski *et al*., 2016; Liu *et al*., 2014; Liu *et al*., 2016; Makino *et al*., 2013; Makino *et al*., 2015; Zinder *et al*., 2016). No clear differences were observed in the patterns of RNA fragment lengths ( Figure 1E).

We also considered the possibility that the failure to clearly detect the expected major protected fragments of ~30 nt might result from insufficient nuclease digestion, leaving fragments with heterogeneous extension beyond the exosome channel. To assess this, the CRAC analysis was repeated for Rrp44-HTP, with 10 fold more RNase A + T1 than normally used. This treatment reduced the relative recovery of the short fragments, but did not generate a clear ~30 nt peak ( Figure 1F). However, a substantial increase in the ~20 nt fragments was revealed. Since these are normalized data, it is unclear whether increased RNase digestion resulted in a higher production of the 20 nt fragment at the expense of longer species, or whether this represents the presence of a certain RNA population in a distinct, highly RNase-resistant RNA-exosome complex.

Notably, inspection of published exosome structural data ( Kowalinski *et al*., 2016; Liu *et al*., 2014; Liu *et al*., 2016; Makino *et al*., 2013; Makino *et al*., 2015; Zinder *et al*., 2016) does not indicate a clear Rrp44-RNA interaction that would be expected to protect an RNA region of this length, suggesting the existence of an additional pathway for RNA to interact with Rrp44.

Almost all of the sequence data analyzed here was generated using “standard” 50 nt Illumina sequencing runs. Since the linker is also sequenced, this limits the effective read length to around 35 nt. We considered the possibility that discrete bands might be seen with longer sequence reads. Indeed, when sequencing was performed with 100 or 150 nt reads, two additional peaks were observed at 39 and 44 nt ( Figure 1G). These were seen with both Rrp44-exo and with Rrp44-exo-S1 double mutation, which disrupts RNA binding by the S1 domain (Rrp44-S1; G916E) as previously reported ( Schneider *et al.*, 2007) and inhibits use of the direct access route for substrates to Rrp44 ( Delan-Forino *et al*., 2017), indicating RNA threading through the central channel of the exosome. They were also seen with preparations at 350 mM and 1M NaCl, apparently precluding protection by the intact TRAMP complex, which is highly salt labile ( LaCava *et al*., 2005). It seems probable that the peaks in read length reflect protection of RNAs that extend through both the exosome core and the RNA helicases Ski2 and/or Mtr4, which bind over the entry pore ( Falk *et al*., 2014; Kowalinski *et al*., 2016; Liu *et al*., 2016; Schmidt *et al*., 2016; Schuch *et al*., 2014).

### Mapping the long and short RNA fragments

**Figure 2.**
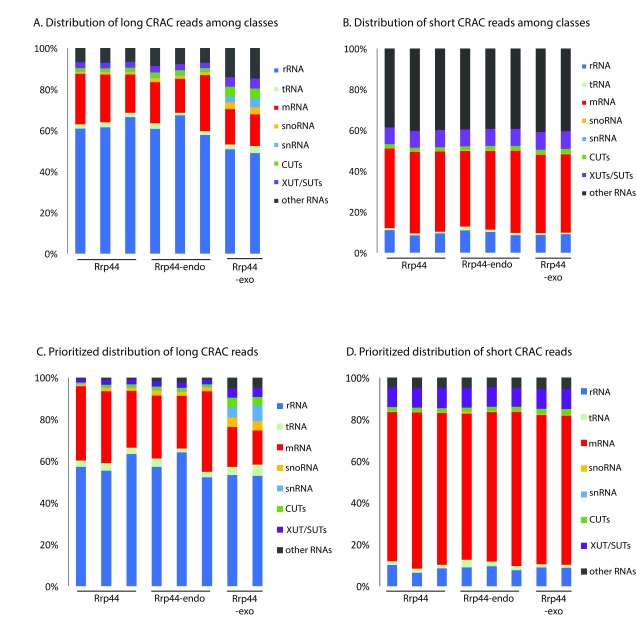
Mapping of long and short reads among RNA classes. ( **A**– **D**) Distributions of long ( **A**, **C**) and short ( **B**, **D**) mapped reads recovered in CRAC datasets between RNA classes, using default counting of overlaps with genes output ( **A**, **B**) or prioritized count ( **C**, **D**). For prioritized alignment, RPKM values were calculated for each long read aligned to the genome, sorted by value and then used as priority order for reads aligning to different places in the genome, to reduce mis-mapping (see Materials and Methods). Two or three biological replicates are shown for each protein.

We anticipated that mapping the short reads to the entire yeast transcriptome would be problematic because any 10 nt sequence is expected to occur more than once in the ~12.1 Mb genome of *Saccharomyces cerevisiae*. The distribution of long reads across the yeast genome was consistent with previous analyses of the sequence data ( Figure 2A), with the greatest number of reads mapping to the pre-rRNA across all datasets. However, short reads were very frequently mapped to regions that do not encode annotated transcripts (included in “other RNAs” in Figure 2), which are generally transcribed at very low levels ( Tuck & Tollervey, 2013). Reads that can be aligned to more than one position in the genome can either be ignored, potentially resulting in a great loss of information, or randomly distributed between the potential targets, as was done in Figure 2. However, it seemed likely that the correct location would be in transcripts that are most frequently bound by the exosome. We therefore prioritized the mapping data, such that transcripts most frequently identified as exosome targets using the long sequence reads, were searched first for matches to the short reads ( Figures 2C and D). This drastically reduced the recovery of reads mapped to non-coding regions, to levels similar to the long reads, strongly suggesting that the reliability of the mapping data had been significantly improved. Note, however, that with any individual, abundant RNA transcript mis-mapping of reads is expected to be much less of a problem using this approach, it is likely that across all mRNAs substantial numbers of reads are still mis-assigned.

**Figure 3.**
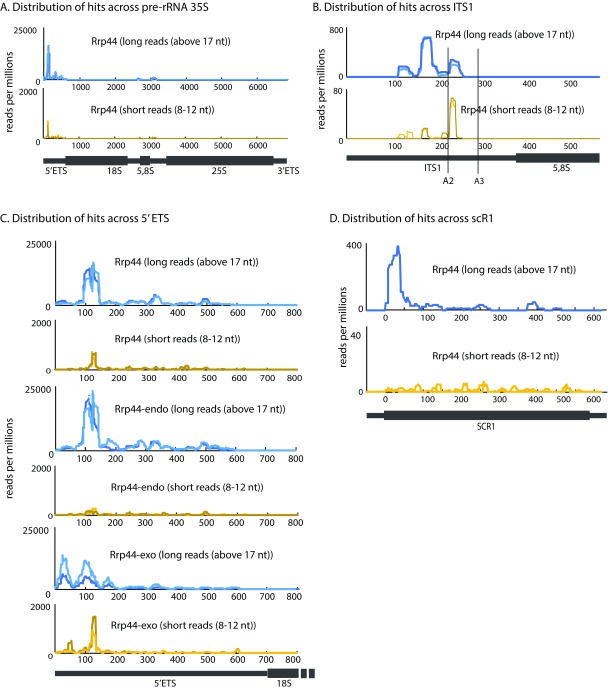
Distributions of long and short reads across pre-rRNA and scR1. ( **A**– **C**) Distributions of long and short reads across the pre-rRNA. ( **A**) Full length 35S pre-rRNA reads recovered with Rrp44. ( **B**) Internal transcribed spacer 1 (ITS1) and 5.8S rRNA reads recovered with Rrp44. ( **C**) 5’ external transcribed spacer (5’ ETS) reads recovered with Rrp44, Rrp44-exo or Rrp44-endo. ( **D**) Distribution of long and short reads recovered with Rrp44, across scR1. Data were normalized by millions of reads. Two biological replicates are shown in each graph. Scale is linear.

The 35S pre-rRNA is a major target for the exosome, but, with or without prioritization of the targets, short reads from all datasets were aligned with the pre-rRNA much less frequently than the long reads. We therefore specifically analyzed the distribution of reads across this 7 kb transcript ( Figures 3A–C). Rrp44 long reads were most frequently recovered from internal transcribed spacer 1 (ITS1) ( Figure 3B) and the 5’ external transcribed spacer (5’ ETS) ( Figure 3C), both of which are subject to exosome-mediated degradation ( Allmang *et al*., 2000). The locations of the long and short reads were in agreement, strongly indicating the latter had been faithfully mapped. However, the proportion of short reads that were mapped to the pre-rRNA was much lower (graphs in Figure 3 show hits per million reads; note differences in scale), indicating that the pre-rRNAs are strongly disfavored substrates for the pathway that generates the short fragments.

Comparison of the locations of long and short reads recovered for Rrp44–endo and Rrp44–exo supported this conclusion (shown for the 5’ ETS region in Figure 3C). We note that the short reads appeared to map towards the 3’ end of peak regions observed for long reads. This strongly indicates that the short fragments are not generated by 3’ degradation of the regions that generate the long reads. In such a case, the fragments would be expected to share 5’ ends. The data would better fit a model in which longer RNA regions are associated with stalled or slowed exosome complexes, giving rise to the peak in occupancy. The short reads are the 3’ fragments of these regions, consistent with their generation by endonuclease cleavage. We note that the region of the 5’ ETS with the greatest exosome occupancy was previously reported to be a target for the endonuclease activity of Rrp44 ( Lebreton *et al*., 2008; Schaeffer *et al*., 2009; Schneider *et al*., 2009).

The distribution of hits along the cytoplasmic RNA component of the signal recognition particle scR1 ( Figure 3D) was different from the 35S pre-rRNA. The high accumulation of Rrp44 at the 5’ end of the RNA was completely lost in the short reads, suggesting that scR1 is targeted independently of the pathway generating the 8-12 nt fragments.

**Figure 4.**
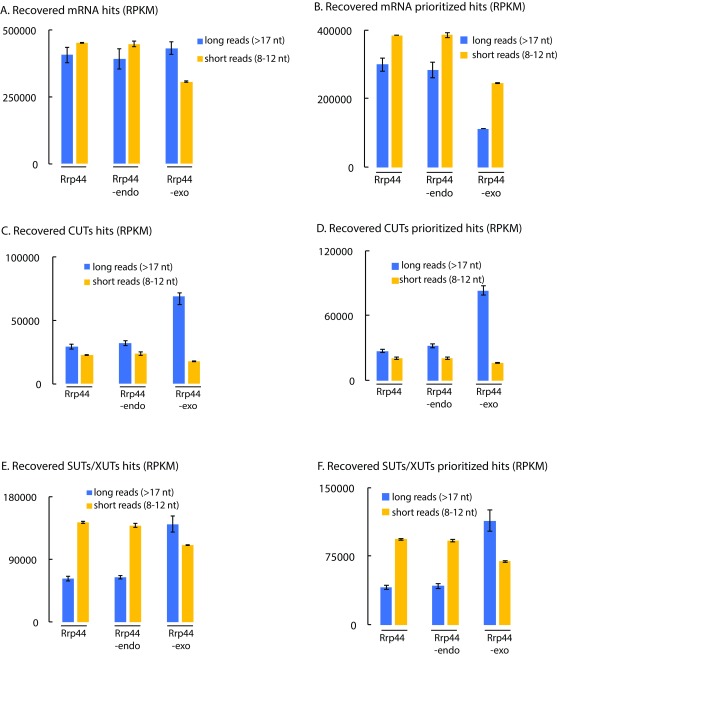
Short reads are preferentially mapped to mRNAs, SUTs and XUTs compared to CUTs. ( **A**– **F**) RPKMs were calculated for Rrp44, Rrp44-endo and Rrp44-exo and summed for all mRNAs ( **A**, **B**), CUTs ( **C**, **D**) and SUTs/XUTs ( **E**, **F**) using default counting of overlaps with genes ( **A**, **C**, **D**) or prioritized counting ( **B**, **D**, **F**). RPKM for long (blue) and short (yellow) reads are averaged between two or three independent experiments and shown with standard deviation.

Since it appeared that the short reads can faithfully be mapped, at least on some transcription units, we assessed their distribution on other major exosome substrates ( Figure 4). On mRNAs, the number of short reads was substantially increased in the prioritized data (panel B) relative to unprioritized (panel A), probably because many more reads are mis-mapped to non-coding regions in the latter. In the prioritized data, it is notable that the relative frequency of short reads mapping to mRNAs was substantially elevated in the short read population, especially for Rrp44-exo. This indicates that mRNAs are preferentially targeted to the direct access route to the Rrp44 exonuclease active site (or preferentially subjected to endonuclease cleavage while threaded to the Rrp44 exonuclease site). Conversely, the CUT class of ncRNAs was strongly disfavored in the short read population in all datasets, but most strikingly for Rrp44-exo ( Figures 4C and D). The significance of this observation was supported by comparison with the SUT/XUT ncRNAs, which are of similar length and expression, but were substantially better represented in the short read population ( Figures 4E and F). The CUTs and SUTs differ strongly in their susceptibility to nuclear RNA degradation and this appears to be reflected in the read length distribution.

## Discussion

Our expectation was that read length analysis would identify a predominant population of ~30 nt species representing RNAs protected by threading through the central channel of the exosome, as previously observed *in vitro* with reconstituted complexes. However, no such peak was observed in any dataset. One possibility was that co-purification of co-factors may consistently result in longer regions of protection. In addition, all datasets unexpectedly showed a broad peak of read length distribution around 20 nt, which was increased by more extensive RNase digestion. Several structural analyses have been reported for exosome complexes *in vitro.* These do not include obvious RNA binding interactions that would give rise to the pattern of RNA protection generated by the *in vivo* derived complex.

The recovered cDNAs also showed a marked peak for shorter reads of 9-12 nt, particularly for Rrp44-exo, which lacks exonuclease activity. This length would fit well with the direct access route to the Rrp44 active site that bypasses the central channel. We expected to find this read-length in the data due to protection in this route. Unexpectedly, however, the peak of short reads was apparently lost for Rrp44-endo, which lacks endonuclease activity, due to point mutations in the PIN domain active site. Separation of the NTD and CTD of Rrp44 using “split” CRAC clearly showed that the ~10 nt fragments are associated with the CTD, which harbors the exonuclease activity, as well as multiple CSD and S1 RNA binding domains. These observations suggest the model that RNAs associated with the Rrp44 CTD can be trimmed to ~10 nt by the activity of the PIN domain endonuclease activity located in the NTD, and that this gives rise to at least some of the short protected fragments recovered.

Rrp44 is a member of the RNase II/RNase R family of processive 3’ exonucleases and, like other family members, can tightly bind the 3’ end of the RNA substrate in an anchoring region ( Frazão *et al*., 2006; Lorentzen *et al*., 2008; Zuo *et al*., 2006). In Rrp44, this anchoring region binds around 9 nt of single-stranded RNA ( Lorentzen *et al*., 2008). This single-stranded RNA-binding pore will contribute substantially to the processivity of RNA degradation by Rrp44, which requires continuous, tight substrate binding between rounds of catalysis. However, it poses a potential problem during RNA processing and degradation *in vivo*. It has long been observed that presumed intermediates in exosome degradation are detectably oligoadenylated by the TRAMP complex, indicating that multiple rounds of degradation and adenylation may be needed for complete degradation of large, highly structured RNA-protein complexes ( Houseley & Tollervey, 2006; LaCava *et al*., 2005). However, re-adenylation requires the substrate RNA to be removed from the exosome channel, an activity that may be slowed or blocked by high-affinity binding of the 3’ end to the anchor site in Rrp44. We speculate that substrate release for re-adenylation may be facilitated by cleavage of the stalled substrate by the Rrp44 endonuclease activity, leading to a ~10 nt fragment remaining associated with the exonuclease domain, and release of the remainder of the substrate for further rounds of TRAMP-mediated tailing and degradation.

Initial structural data on the RNA-bound exosome complex indicated that the PIN domain active site is exposed to the solvent rather than the lumen of the exosome ( Makino *et al.*, 2013). However, subsequent analyses indicated that the exosome can undergo conformational changes that potentially open a route from the central channel to the endonuclease active site ( Han & van Hoof, 2016; Liu *et al.*, 2014; Makino *et al.*, 2015). This model is supported by biochemical analyses indicating that the efficiency of endonuclease activity of the exosome is dependent on the central channel ( Wasmuth & Lima, 2012; Zinder *et al.*, 2016). It therefore seems possible that the endonuclease activity may act on threaded substrates under some circumstances.

A major difficulty in further analyzing the sequence data lies in mapping the ~10 nt RNA fragments to the genome. The yeast genome is around 12.1 Mb, with a potential transcriptome approximately twice this size. In consequence, sequences need to be greater than 12 nt to be expected to identify a unique site in the yeast transcriptome (4 ^12^ = 17 × 10 ^6^). Mapping of ~10 nt fragments therefore creates significant problems with false positive results. Despite this we were able to identify sites where the long and short read populations yielded very consistent mapping data. Prioritization of the data, such that ambiguous reads are first mapped to the most common exosome substrates, appeared to substantially improve the quality of mapping. This provided clear evidence that some substrates are strongly disfavored in the short reads. This was most marked for the pre-rRNAs, which are normally the predominant exosome substrate. Recent data indicated that the exosome-associated RNA helicase Mtr4 is actively and specifically recruited to pre-rRNAs ( Thoms *et al*., 2015), potentially reducing problems due to stalling of these substrate in the channel of the exosome.

A potential approach for further analysis would seem to lie in the assembly of larger contiguous fragments from multiple short reads. However, we have so far been unable to usefully achieve this. Readers who believe they can address this problem are encouraged to re-analyze the sequence data or contact the authors.

## Materials and methods

### Materials and availability of data

Most of the primary sequence data were previously published and deposited in NCBI Gene Expression Omnibus (GEO, http://www.ncbi.nlm.nih.gov/geo/) (RRID:SCR_005012). Rrp44 and Rrp44-exo CRAC datasets were previously published ( Turowski *et al*., 2016) (GEO accession number GSE77863). Rrp44-exo split-CRAC and Rrp44-endo CRAC datasets were previously published in ( Schneider *et al*., 2012). Since one of the two Rrp44-endo-HTP CRAC experiments had a relatively low number of reads, we performed a new CRAC experiment for this mutant (GEO accession numbers GSE40046 and GSE94889).

### CRAC

CRAC was performed as previously described ( Granneman *et al*., 2009; Granneman *et al*., 2011) on yeast strains expressing the protein of interest tagged with a C-terminal HTP tag (His _6_ - TEV protease cleavage site – 2 copies of the Z-domain of protein A), grown in SD-medium to log phase and UV crosslinked (254 nm, 100 sec) to covalently bind RNA to protein. Cells were lysed in buffer containing 50 mM Tris-HCl pH 7.8, 1.5 mM MgCl _2_, 150 mM NaCl, 0.1% NP-40 and 5 mM β-mercaptoethanol, and RNA-protein complexes were isolated by binding to an IgG column. Bound material was washed briefly in the same buffer, but with 1M NaCl (except for the “low salt’ sample in Figure 1E, where 350 mM NaCl was used), followed by more extensive washes in the same buffer containing 150 mM NaCl, and exosome complexes were released by TEV elution. RNAs were partially digested to leave only the “footprint” of the protein or protein complex using RNaceIT Ribonuclease Cocktail (Agilent) (for Figure 1F, 10X RNase treatment was used). Subsequently, the proteins were denatured by incubation with 6M Guanidinium HCl prior to binding to a nickel affinity column. Linker ligation (Mircat linkers and barcoded linkers were ligated on the 3’ and 5’ ends, respectively) and radiolabeling of the crosslinked RNA fragments was performed on the nickel column. Bound proteins were eluted with imidazole and further purified by denaturing SDS polyacrylamide gel electrophoresis (SDS-PAGE) on NuPage 4–12% gradient gels with Bis-TRIS buffer. This gel system is used since the pH remains roughly 7.0 during the run. In more commonly used SDS-PAGE protocols, the pH can rise to 9, leading to RNA hydrolysis. Protein-RNA complexes were transferred to nitrocellulose, identified by autoradiography, and excised.

In one set of replicate experiments, the barcoded Rrp44-exo, Rrp44-endo and WT control samples were mixed following elution from the nickel column. In the other replicate, the samples were handled in parallel. In neither case would differences in the regions excised from the SDS-PAGE protein gel/nitrocellulose membrane, or subsequent agarose gel with the PCR products, give rise to the observed differences in cDNA length profiles.

The proteins were then digested with proteinase K and the associated RNAs amplified by RT-PCR, as previously described ( Tuck & Tollervey, 2013) using PCR primer PE: GCAGAAGACGGCATACGAGATCGGTCTCGGCATTCCTGGCCTTGGCACCCGAGAATTCC; and PCR primer P5: AATGATACGGCGACCACCGAGATCTACACTCTTTCCCTACACGACGCTCTTCCGATCT. cDNA libraries were size fractionated on agarose gel and then subjected to next-generation sequencing using Illumina Hi-Seq (Edinburgh Genomics) or Illumina Miniseq (our laboratory). In one set of replicate experiments, the barcoded Rrp44-exo, Rrp44-endo and WT control samples were mixed following elution from the nickel column. In the other replicate, the samples were handled in parallel.

### Sequencing data analysis

***Pre-processing and alignment.*** Sequencing data were quality filtered and adapters were trimmed using Flexbar 2.5 ( Dodt *et al*., 2012) with parameters –at 1 –ao 4 and only reads containing the 3’ adapter were retained. For all alignments, sequences shorter than 8 nt or considered as low complexity (reads having more than 75% of their content corresponding to a single nucleotide stretch and that would be potentially misaligned) were filtered out. Reads were then aligned to the *S. cerevisiae* genome (SGD v64) using Novoalign (V2.07.00, Novocraft) with genome annotation from Ensembl (EF4.74) ( Flicek *et al*., 2014), supplemented with non-coding sequences as described in ( Tuck & Tollervey, 2013), with parameters –r Random, -l 8. For each sample, either mapped reads equal to or longer than 17 nt, considered as “long reads”, or reads between 8 and 12 nt, considered as “short reads” were selected and processed separately in downstream analyses.

***Counting overlaps with features and prioritization.*** Downstream analyses were performed using pyCRAC software ( Webb *et al*., 2014). To count overlaps with genes and reads per millions per kilobase (RPKM), pyReadCounters (pyCRAC package) was used. Substantial numbers of short reads were aligned to antisense features, which we assumed was mainly mis-mapping due to the ability of a single short read to align to different features. To reduce mis-mapping, we chose to prioritize mapping to well-represented features over targets recovered with low frequency. For this, the RPKM for each single feature was calculated from alignments of long reads. Features were then sorted by RPKM value and the output list used as a priority order. In particular, antisense RNAs were given lower priority than any other genomic feature, since previous strand-specific mapping of RNAPII demonstrated their low expression ( Milligan *et al*., 2016). Overlaps with genes for short and long reads were then calculated again using this priority list and a single read aligning to two or more features was counted as mapping to the highest ranked gene.

### Plots, binding profiles

Plots showing binding along single genes were generated using pyPileup (pyCRAC package) and normalized per reads per millions.

## Data availability

All sequence data are available from GEO (RRID:SCR_005012) under accession numbers GSE77863, GSE40046 and GSE94889.
