# Supplementary material for: RNA substrate length as an indicator of exosome interactions in vivo
Source: Wellcome Open Res. 2017 Jul 3;2:34. Originally published 2017 May 25. [Version 2] doi: 10.12688/wellcomeopenres.10724.2 (PMC5500899; doi:10.12688/wellcomeopenres.10724.2)
Supplement: Peer review history

# Peer review: Referee response for version 2

**DOI:** 10.21956/wellcomeopenres.12970.r23991

**Published:** 2017-07-06

**Authors:** Hongwei Wang

**Affiliations:** 1 School of Life Sciences, Tsinghua University, Beijing, 100084, China

The revised version has addressed most of the questions raised by the reviewers. Certainly new experiments can be performed in the future to reveal the exosome-RNA interactions more thoroughly.

I have read this submission. I believe that I have an appropriate level of expertise to confirm that it is of an acceptable scientific standard.

# Peer review: Referee response for version 1

**DOI:** 10.21956/wellcomeopenres.11564.r23179

**Published:** 2017-06-20

**Authors:** Hongwei Wang

**Affiliations:** 1 School of Life Sciences, Tsinghua University, Beijing, 100084, China

The exosome complex is an important RNA degradation and processing machinery that is responsible to degrade RNAs from their 3’ ends. Previous works suggested the presence of at least two routes for RNA substrates to be recruited to the exosome complex’s exonuclease activity site in the Rrp44/Dis3 protein. The Rrp44 protein also has a weak endonuclease activity site in its N-terminal region. The activity of exosome is also regulated by a few co-factor proteins. These all lend the complex a rather complicated RNA degradation or processing mechanism that still awaits to be fully revealed.

 The authors in this work used CRAC technology in combination with the RNase protection to analyze the RNA substrates that are loaded to Rrp44 protein and protected by the exosome complex. Their results showed the accumulation of 10-nt RNA species bound to Rrp44’s C-terminal region and the absence of 30-nt RNA species. Instead, they observed a broad shallow peak around 20-nt. Interestingly, the lack of endonuclease activity caused the reduction of the 10-nt RNA species. Deep sequencing analysis of the bound RNAs showed a whole spectrum of RNA species bound to Rrp44, indicating a complex behavior of the exosome in RNA substrate recruitment in vivo. The authors proposed the presence of another pathway for RNA substrates to be recruited to the exosome and a potential role played by the endonuclease during the different pathways. This work provided new data to study the exosome-RNA transactions although the exact mechanism seemed more complicated than proposed previously or in this work.

 More data would be helpful if the authors can also perform similar CRAC of yeast strains lacking Rrp6 gene to understand the cytoplasmic exosome’s substrate more clearly. A minor issue is that the author didn’t explain what the Rrp44-exo-S1 mutant is.

I have read this submission. I believe that I have an appropriate level of expertise to confirm that it is of an acceptable scientific standard, however I have significant reservations, as outlined above.

# Peer review: Referee response for version 1

**DOI:** 10.21956/wellcomeopenres.11564.r23017

**Published:** 2017-06-19

**Authors:** Domenico Libri

**Affiliations:** 1 Institut Jacques Monod, Paris Diderot University, Paris, France

In this article the authors analyze the RNA fragments that associate *in vivo* with the wild type exosome or with mutants that are defective for the exo- or endonucleolytic function.  They exploit the RNase protection step included in the CRAC protocol and assess the average length of protected fragments to infer the topology of RNA degradation by the exosome . Using a standard protocol for sequencing (50nt reads) they see a distribution of length with a clear peak at very short reads (6-8 in the wt and 9-10 in the exo- mutant). Formation of these short fragments requires the endonucleolytic function of the exosome. The authors propose that these fragments derive from endonucleolytic cleavage of stalled substrates in the central channel of the exosome.

 Using a longer read sequencing protocol, they observed the presence of two additional peaks (39 and 44nt) that likely represent fragments derived from substrates that are being degraded after threading through the central channel. Mapping of these read classes allowed inferring on the substrate preferences for the mechanisms of degradation (i.e. direct path or threading through the central core).

 This is a well-conducted study that provides many interesting details on the mechanism of RNA degradation by the exosome. The data convincingly support the model proposed. What follow are a few suggestions that could complement the study.

  

- The distribution of the mismatches due to crosslinking is not exploited in the study. This could provide useful information on the nature of the reads. For instance, the authors propose that the 39 and 44nt reads derive from protection either by the sole central channel, or by the central channel and the helicases associated (Mtr4 or Ski2). If this is true, the two families of reads should have an identical distribution of crosslinking sites when aligned to the 3’ end. Also, reads derived from threading or direct access should have a different distribution of mismatches.
- The authors propose that stalled substrates threaded through the central core are cleaved by the endo activity for further processing. This should imply endo cleavage of substrates within the central core. Is this consistent with current structural models ? The authors should comment on this.
- Use of a mutant that prevents threading might allow demonstrating that the 39nt and 44nt reads indeed derive from substrates being threaded through the central core

I have read this submission. I believe that I have an appropriate level of expertise to confirm that it is of an acceptable scientific standard.

# Peer review: Referee response for version 1

**DOI:** 10.21956/wellcomeopenres.11564.r23177

**Published:** 2017-06-08

**Authors:** Roy Parker

**Affiliations:** 1 Department of Chemistry and Biochemistry, University of Colorado Boulder, Boulder, CO, USA

This manuscript addresses the paths by which RNAs enter the exonucleolytic and/or endonucleolytic active sites of the Rrp44, which is the catalytic subunit of the eukaryotic exosome. This is an interesting issue since the exosome has two main nucleolytic activities and their relative importance and how different RNAs access these different sites is not clear. Based on *in vitro* experiments, one would predict that the exosome would protect RNAs of ~30 bases when they are threaded through the barrel of the exosome, and ~10 bases when the RNA accesses the active site directly.

 The main contribution of this work is to use deep sequencing to identify fragments of RNA protected by the exosome *in vivo*.  The approach is to cross link RNA to the exosome *in vivo*, purify the exosome and then the cross-linked RNAs are digested to protected fragments subjected to deep sequencing.  This work revealed the WT exosome protects a broad range of RNA sizes with three main classes: 1) an enrichment ~6-8 bases, which is dependent on the endo activity, and converts to ~10 bases in the absence of exo activity: 2) a broad size distribution from 12-34 that is largely independent of exo or endo nuclease activities, and 3) RNAs of 39 and 44, which are not dependent on the direct entry channel and are likely to reflect RNAs that are threaded through the exosome barrel, but more than 30 bases are protected possibly due to exosome co-factors.

 Taken together, this provides insight that the endonuclease activity may be quite important for releasing RNAs that are stalled during exonucleolytic degradation.  It also highlights that understanding the size of RNAs protected by the exosome *in vivo* will suggest unanswered questions about how RNA molecules interact with the exosome and access the active sites.

 The experiments are well done and the interpretations valid.  Some suggestions that could be considered to improve the work are given below:  

  

1. Although not required for indexing, it would be interesting to know if the reads that map to mRNAs (Figure 2C&D) map to distinct subsets of mRNAs. For example, the exosome would be predicted to preferentially target mRNAs with slow decapping rates, which might be revealed by a more granular analysis of the read distribution to different mRNAs.
2. I might rephrase the summary of the work to highlight that there is a clear set of fragments of 39 & 44 bases, which would be consistent with RNAs threaded through the barrel of the exosome but protected by additional co-factors.  As written now, one is not aware of these fragments till deeper in the manuscript and the comments about co-factors in the conclusions come a bit out of the blue.
3. Although not required for indexing, it would be interesting to see how the shorter reads are affected by the exo-S1 mutations. 

I have read this submission. I believe that I have an appropriate level of expertise to confirm that it is of an acceptable scientific standard.
